# Retraction: High-performance sono/nano-catalytic system: CTSN/Fe_3_O_4_–Cu nanocomposite, a promising heterogeneous catalyst for the synthesis of *N*-arylimidazoles

**DOI:** 10.1039/d4ra90044h

**Published:** 2024-04-24

**Authors:** Reza Taheri-Ledari, Seyed Masoud Hashemi, Ali Maleki

**Affiliations:** a Catalysts and Organic Synthesis Research Laboratory, Department of Chemistry, Iran University of Science and Technology Tehran 16846-13114 Iran maleki@iust.ac.ir +98 21 73021584 +98 21 73228313

## Abstract

Retraction of ‘High-performance sono/nano-catalytic system: CTSN/Fe_3_O_4_–Cu nanocomposite, a promising heterogeneous catalyst for the synthesis of *N*-arylimidazoles’ by Reza Taheri-Ledari *et al.*, *RSC Adv.*, 2019, **9**, 40348–40356, https://doi.org/10.1039/C9RA08062G.

The Royal Society of Chemistry hereby wholly retracts this *RSC Advances* article due to concerns with the reliability of the data reported in Fig. 2B of the article and the NMR spectra in the supporting information.

Fig. 2b of this article is identical to Fig. 4b in ref. 1. The authors have stated that there was an error in ref. 1 and that this article does not need a correction.

An independent expert has reviewed both this article and ref. 1 and has raised concerns with the NMR data in the electronic supplementary information (ESI). Several of the NMR spectra have strange features, for example baselines with small gaps and a lack of noise in the baseline.

The authors have not been able to provide the original raw NMR data so we are unable to verify the reliability of the published data.

Given the significance of the concerns about the validity of the data and the lack of raw data, the findings presented in this paper are not reliable.

The authors were informed about the retraction of the article. Reza Taheri-Ledari has agreed with the decision, the other authors have not responded.

Signed: Reza Taheri-Ledari

Date: 10th April 2024

Retraction endorsed by Laura Fisher, Executive Editor, *RSC Advances*

## Supplementary Material
